# Biofabrication of Artificial Stem Cell Niches in the Anterior Ocular Segment

**DOI:** 10.3390/bioengineering8100135

**Published:** 2021-09-30

**Authors:** Veronica Hidalgo-Alvarez, Hala S. Dhowre, Olivia A. Kingston, Carl M. Sheridan, Hannah J. Levis

**Affiliations:** 1 Institute of Biological Chemistry, Biophysics and Bioengineering, School of Engineering and Physical Sciences, Heriot-Watt University, Edinburgh EH14 4AS, UK; 2 Department of Eye and Vision Science, Institute of Life Course and Medical Sciences, University of Liverpool, Liverpool L7 8TX, UK; h.s.dhowre@liverpool.ac.uk (H.S.D.); olivia.kingston@liverpool.ac.uk (O.A.K.)

**Keywords:** ophthalmic bioengineering, stem cells, biofabrication, cornea, trabecular meshwork, conjunctiva, regenerative medicine, tissue engineering, stem cell niche, in vitro 3D models

## Abstract

The anterior segment of the eye is a complex set of structures that collectively act to maintain the integrity of the globe and direct light towards the posteriorly located retina. The eye is exposed to numerous physical and environmental insults such as infection, UV radiation, physical or chemical injuries. Loss of transparency to the cornea or lens (cataract) and dysfunctional regulation of intra ocular pressure (glaucoma) are leading causes of worldwide blindness. Whilst traditional therapeutic approaches can improve vision, their effect often fails to control the multiple pathological events that lead to long-term vision loss. Regenerative medicine approaches in the eye have already had success with ocular stem cell therapy and ex vivo production of cornea and conjunctival tissue for transplant recovering patients’ vision. However, advancements are required to increase the efficacy of these as well as develop other ocular cell therapies. One of the most important challenges that determines the success of regenerative approaches is the preservation of the stem cell properties during expansion culture in vitro. To achieve this, the environment must provide the physical, chemical and biological factors that ensure the maintenance of their undifferentiated state, as well as their proliferative capacity. This is likely to be accomplished by replicating the natural stem cell niche in vitro. Due to the complex nature of the cell microenvironment, the creation of such artificial niches requires the use of bioengineering techniques which can replicate the physico-chemical properties and the dynamic cell–extracellular matrix interactions that maintain the stem cell phenotype. This review discusses the progress made in the replication of stem cell niches from the anterior ocular segment by using bioengineering approaches and their therapeutic implications.

## 1. Introduction

**Figure 1:**
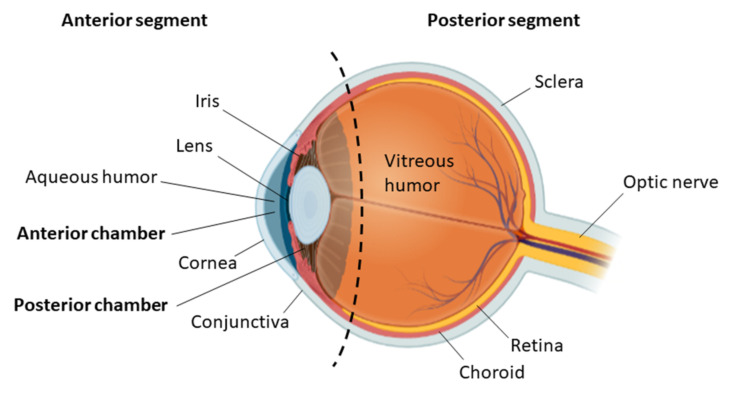
Anatomy of the vertebrate eye. This organ is divided into the anterior and posterior segments. The anterior segment comprises the anterior chamber, which is located between the cornea and the iris, and the posterior chamber, which extends from the iris to the lens. Adapted from Lin et al. (2019) [21].

The eye is a complex organ that allows the visual perception of the environment by capturing the light reflected by the surrounding objects and transforming it into electrical signals that can be recognized and processed by the brain. This phenomenon requires a fine coordination between the different tissues that constitute the eye (Figure 1), as each of them plays a key role in this process [1,2]. The first tissue that the light encounters is the cornea, which refracts the light rays before being transmitted through the pupil into the lens [3]. Depending on the availability of light, the iris contracts or expands in order to adjust the pupil size. This regulates the amount of light that reaches the lens [4]. Whilst most of the refractive power is provided by the cornea, the function of the lens is to cause small changes in refraction in order to focus objects that are located at different distances [5]. This is achieved by adjusting the lens shape by means of contraction and relaxation of the ciliary muscle [6]. After passing through the lens, the light is focused onto the focal point situated in the retina [1]. This tissue is formed of different types of photoreceptors, each of them sensitive to light of a particular wavelength and intensity [7,8]. These cells have the ability to convert the incident light into electrical signals in a process that involves the conformational change in retinal, which is an aldehyde of vitamin A. A series of enzymatic reactions is subsequently activated, resulting in hyperpolarisation. The electrical signals generated in this process are then transmitted via neuronal synapses into the optic nerve, which carries this information to the visual cortex in the brain [9].

The maintenance of a healthy status in every single constituent of the ocular globe is essential for the preservation of the individual’s visual quality. However, this is particularly difficult in the anterior segment of the eye, which is constantly exposed to environmental insults such as UV radiation, injuries or infections. The treatment of the wide range of diseases that result from such stresses poses a significant challenge for clinicians, as conventional therapies have been generally unsuccessful in curing such syndromes. On the other hand, reconstructive therapies based on the use of stem cells hold great promise for treating ocular diseases [10].

Stem cells are relatively undifferentiated cells that have the ability to self-renew and generate one or more differentiated progeny cells [10,11,12]. In embryos, stem cells give rise to cells from almost all tissues in the body by responding to positional cues that trigger differentiation to specific cell lineages [13]. Alongside differentiated cells, embryonic stem cells (ESCs) generate progenitor cells and adult stem cells, whose differentiation potential is more constrained than that of ESCs [14]. In adults, the function of these cells is to replenish any cells that are lost or damaged in the tissue where they reside [15]. Adult stem cells can be found in reservoirs known as stem cell niches, which are located in many tissues and organs throughout the body. Such microenvironments ensure the maintenance of the stem cells’ undifferentiated state and multipotency [16].

Given the heterogeneity of the tissues that comprise the ocular globe, different stem cell niches can be found in this organ [17]. The replication of such environments in vitro is one of the decisive factors that determine the success of stem cell therapy, as cells need to receive the physical, chemical and biological factors that maintain their properties prior to implantation in patients [10,18]. The advent of bioengineering techniques in recent years has facilitated the development of artificial microenvironments that can recreate the native stem cell niche. As well as being used for the maintenance of an undifferentiated state in stem cells that are to be used in stem cell therapy, bioengineered cell niches can be used as in vitro models to study the niche homeostasis or to evaluate the effect of therapeutic agents on these environments. Furthermore, they can act as prosthetic artificial constructs that restore damaged ocular tissues in order to cure visual impairment or loss [19,20].

This review describes the advancements that have been made in the replication of each of the stem cell microenvironments that are located in the anterior segment of the vertebrate eye. The biofabrication methods employed in their synthesis and their therapeutic implications are discussed in detail.

## 2. Ocular Disease and Stem Cell Therapy

Blindness or vision loss can be caused either by the failure of the light to reach the retina or the inability of the retina to convert the light into electrical signals [22]. The main factors that cause this disability are physical trauma or diseases such as cataract, age-related macular degeneration or glaucoma [23]. The anterior segment of the eye, which is formed of the anterior and posterior chambers, is particularly vulnerable to such pathologies as a consequence of its direct exposure to environmental and physical stresses. While conventional therapies such as drug administration, surgery or laser treatment can improve the visual quality temporarily, they have not proven to be efficient in preventing the progression of blindness [24,25]. Conversely, stem cell therapy has shown great potential to treat ocular diseases by contributing to the restoration of any damaged tissue. Due to the accessibility of the eye and its immune-privileged status, ocular stem cell therapy is a rather common procedure, the cornea being one of the most targeted tissues for this type of therapy [26].

Different types of stem cells are currently available for use in ocular regeneration. These include embryonic stem cells (ESCs), induced pluripotent stem cells (iPSCs) and adult stem cells. While the use of ESCs and iPSCs is highly advantageous due to their pluripotency, it also presents several challenges, such as the potential immune rejection of allogeneic cells, the difficulty to purify a specific cell type from the heterogeneous stem cell-derived population and their tumorigenic potential. These issues can be circumvented with the utilisation of adult stem cells, which allow for autologous cell therapy and have a lower tumorigenic potential than ESCs and iPSCs [12,18]. As previously mentioned, adult stem cells reside in niches where they are continuously generated [27]. This is essential for the maintenance of the tissue homeostasis and the regeneration of damaged structures [10]. The ocular anterior segment contains a variety of stem cell niches with different stem cell populations for the restoration of each of the components of this part of the eye (Table 1). The replication of such environments in vitro is essential for the survival of stem cells while they are in expansion prior to implantation in patients. The following sections describe in detail each of the stem cell niches located in the anterior ocular segment and discuss the progress made in the bioengineering of these cell microenvironments.

## 3. Bioengineered Stem Cell Niches

**Figure 2:**
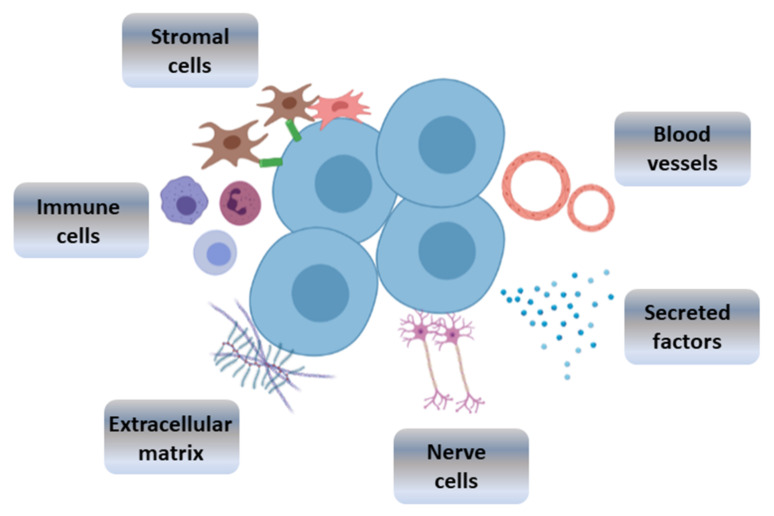
Schematic diagram of the stem cell niche showing the stem cells and the different components that interact with them: stromal cells, immune cells, extracellular matrix, nerve cells and signalling factors that are secreted by neighbouring cells or tissues. Blood vessels, which provide nutrients to the niche, are also depicted. The niche components are not drawn at scale. Adapted from Pennings et al. (2018) [52].

Stem cell niches are specialised and controlled local tissue compartments that contribute to the maintenance of multipotent stem cell populations in adult tissue [47]. They provide the conditions and factors required to preserve the proliferative potential and multipotency of stem cells [48]. Stem cell niches are formed of extracellular matrix (ECM) and support cells, such as mesenchymal or stromal cells, that establish interactions with stem cell and provide them with paracrine signals (Figure 2). Stem cells and their progeny can also act as support cells by providing autocrine and paracrine regulatory factors. Some of the signalling pathways involved in stem cell homeostasis include TGF-β, BMP, Notch and Wnt [16,49]. Cell–cell and integrin-mediated cell–ECM adhesions are crucial for the maintenance of the niche architecture, the generation and transmission of signals and the control of the stem cell divisions. Stem cells located in niches undergo either symmetric divisions that lead to an augmentation of the stem cell population or asymmetric divisions that result in the maintenance in the number of multipotent stem cells and the generation of progenitor cells. The frequency of these divisions and the egression of progenitor cells from the niche are finely controlled by this microenvironment [50].

The maintenance of stem cells requires a coordinated interaction between physical, chemical and biological factors. Physical cues include the ECM elasticity and stiffness, which can regulate stem cell differentiation to different lineages [16]. Sources of chemical and biological signals include blood vessels, neurons and immune cells, which are also considered key components of the stem cell niche [16,49]. Furthermore, stem cell niches are dynamic environments that are continuously remodelled in order to achieve homeostasis [20]. This adds another level of complexity that makes it difficult to achieve a deep understanding of the niche structure and the processes that take place in these environments. Therefore, the development of artificial analogues is highly advantageous for the study of stem cell niches. Stem cell therapy would also benefit directly from the development of bioengineered microenvironments, as they would lead to an increase in stem cell survival prior to implantation [51].

The replication of stem cell microenvironments is highly challenging and requires the use of biofabrication techniques. This involves the use of biomaterials that mimic the nanoscale architecture and chemical composition of the ECM and the incubation in bioreactors which recreate the physiological conditions [20,51]. The mechanical strength of the biomaterials, the establishment of oxygen gradients and the presence of growth factors are other characteristics that have to be considered when producing an artificial microenvironment. Furthermore, such constructs must have a high bioactivity in order to allow the establishments of functional interactions with the stem cells [19,20].

A wide variety of biofabrication techniques are available for the production and functionalisation of bioengineered constructs. Some of these methods are capable of creating 3D structures with controlled geometries and physico-chemical properties. Furthermore, these technologies allow the integration of biomacromolecules in order to increase the bioactivity of the resulting structures [53]. Some of the biofabrication methods that are currently available for the creation of stem cell niches are explained below and summarised in Table 2.

### 3.1. Photolithography

The working principle of photolithography techniques is based on the use of light to transfer geometric shapes or patterns to a photosensitive material by means of a photomask. The popularity of this method in tissue engineering is due to the uniform cell encapsulation that can be achieved, the reduced heat production and the high degree of control over the reaction kinetics. However, the photoinitiator molecules that are present in the formulations can generate toxic free radicals [53].

### 3.2. Electrospinning

Electrospinning is a versatile method to produce of nanofibers by applying a high voltage to a polymer solution as it passes through a needle. The adjustment of parameters such as the applied voltage, flow rate or polymer concentration results in the generation of fibres with controlled size and morphology. Some of the advantages of the use of this technique include the wide variety of polymers that can be processed with it, as well as the relatively low cost and simplicity of the equipment required [54,55]. An additional benefit of using this technology to produce of bioengineered tissues is the high level of biomimicry achieved, as the structure of the resulting nanofibers mimics the architecture of the native ECM [55]. Furthermore, due to their high porosity and surface-to-volume ratio, electrospun fibres enhance cell adhesion, migration and proliferation. However, the high density at which these fibres are packed and the fact that they form two-dimensional structures (2D) rather than three-dimensional (3D) constructs results in a lower degree of cell infiltration, which could be detrimental for tissue engineering applications. To overcome this issue, different approaches have been developed to increase the pore size of the nanofibrous matrix. These include the incorporation of microfibers, the application of salt-leaching methods or the ultrasonication of the nanofibers [55,56,57].

### 3.3. 3D Printing

Three-dimensional printing, also known as additive manufacturing, is a technology that fabricates objects by depositing materials layer by layer until a 3D structure is formed. Before printing, the structure is modelled on computer-aided design software (CAD) or obtained via computerised tomography (CT) scan. This method allows the fabrication of biomimetic structures with specific designs [54,58,59]. Furthermore, the development of 3D bioprinting technologies has enabled the production of cell-loaded constructs that can incorporate growth factors in their formulation. This results in the fabrication of bioengineered constructs that closely resemble the natural tissues or organs. Other biomedical applications of 3D printing technology include the fabrication of models for surgery, drug screening and delivery systems and biosensors. Some of the advantages of using 3D printing for these purposes include the ability to create patient-specific medical devices and the higher resolution and reproducibility in comparison with other approaches [60,61]. While these benefits make 3D printing a very promising approach, it also has some pitfalls that need to be considered when using it. These include the need for 3D design software, long production times when fabricating large constructs and the difficulties encountered when developing printable biomaterials that are biocompatible [61].

All these techniques have been employed in the development of artificial stem cell microenvironments from the anterior surface of the eye. The following sections provide a revision of the advancements made to date on the replication of each of these niches by making use of such technologies.

**Table 2:** Comparison between the different bioengineering techniques employed in the manufacture of ocular stem cell niches.

Technique	Principle	Advantages	Disadvantages	Ref.
3D printing/ bioprinting	Production of 3D structures by depositing materials layer by layer	Production of hydrated polymeric structures; production of custom-built structures; easy integration in the clinic; improved mimicry of anatomical structures; higher resolution and reproducibility	High cost of some types of 3DP equipment; it can take a long time, which is very detrimental when producing large constructs; lower dimensional accuracy with some 3DP techniques; difficulties encountered in the development of printable biomaterials	[54,61,62]
Electrospinning	Production of nanofibers by applying a high voltage to a polymer solution as it passes through a needle	High-scale production; good mechanical properties; Possible surface modification; large surface-volume ratio; nanotopographical control of cells; high versatility in materials (polymers, metals, ceramic)	Difficult to produce scaffolds with a high volume; non-uniform cellular distribution and poor cell infiltration; limited to polymers; inferior macroscopic mechanical properties in comparison with other techniques; possible organic solvent residue	[54,55,63,64]
Photolithography	Use of light to transfer a geometric pattern to a photosensitive material by using a photomask	Uniform cell encapsulation; reduced heat production; controllable reaction kinetics	Potential toxicity of the photoinitiator molecules present in photocurable resins	[53]

## 4. Cornea

The cornea is the avascular, multi-layered and transparent conical shaped outer structure of the eye and it is found anterior to the iris and pupil. The cornea is essential for maintaining vision as it provides approximately 65–75% of the refraction and transmission of light rays to the lens and focuses light on the retina. As the outermost structure, the cornea is mechanically tough as it acts as the physical barrier protecting the inner content of the eye against infection and environmental damage along with the tear film [33,65]. The tear film consists of an outer lipid layer and an inner water-mucous layer that bathe the ocular surface and protect it from chemical, toxic or foreign body damage, microbial invasion and also acts to smooth out micro-irregularities of the ocular surface. The functions of the cornea are governed by its hierarchical structure, which comprises three cell layers and two membranes that are optically transparent: (i) the epithelium, (ii) Bowman’s membrane, (iii) the stroma, (iv) the Descemet’s membrane and (v) the endothelium, with each layer differing in the composition and the type of cells resident (Table 3) [66].

As one of the most densely innervated tissues in the body, the corneal nerves are crucial in regulating the protection and maintenance of the ocular surface integrity as well as being important indicators (i.e., sensory, reflex and trophic functions) of a healing cornea after surgery [67,68]. The health of the stratified ocular surface is fundamental in the function of the cornea, and this is entirely reliant on the niche of stem cells located in the limbal region at the periphery of the cornea. These limbal stem cells divide and differentiate into corneal epithelium over an individual’s lifetime. Thus, they are responsible for healing and regeneration in the event of corneal epithelium injury [26,69,70].

**Table 3:** The hierarchical structure of the cornea.

Cornea Layer	Function	Composition	Cell layers	Types of Cells	Thickness (µm)	Regenerates	Ref.
Epithelium	Barrier to water, microbes and chemicals Performs immunological function through Langerhans cells Provides smooth optical surface to contribute to refractive power of the eye	Stratified squamous epithelium	5 to 7	Cellular (superficial cells) Cellular (wing cells) Cellular (basal cells)	50	Yes (every 7 to 10 days)	[71,72,73,74]
Bowman’s Layer	Involved in maintaining the corneal shape	Compact layer of unorganised collagen fibres	Monolayer	Acellular (collagens (Type I, V), proteoglycans)	12	No	[71,74,75,76]
Stroma	Provides mechanical strength to cornea Involved in the transparency of cornea Acts optically as the main refractive lens	Orderly arrangement of collagen lamellae with keratocytes	200–250 distinct lamellae	Cellular (keratocytes) Acellular (collagens (Type I, II, V, VII), glycosaminoglycans, matrix metalloproteinases)	500	Yes (slow process over several years)	[71,73,74,76]
Descemet’s Membrane	Responsible for anchoring the endothelium to the cornea	Basement membrane materials	Monolayer	Acellular (collagen (Type IV), laminin)	3–10	Yes	[71,74,75,76]
Endothelium	Maintains corneal clarity by pumping water from the stroma Maintains the cornea’s deturgescence via ionic pumps	Single layer of simple squamous epithelium	Monolayer	Cellular	5	No	[73,77,78]

### 4.1. The Corneal Stem Cell Niche

**Figure 3:**
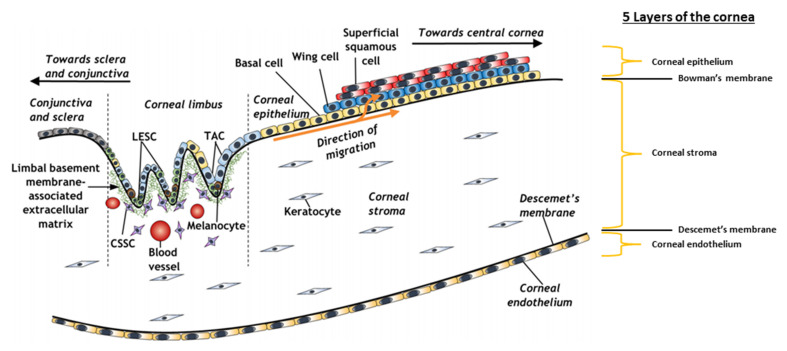
Schematic representation of the corneal limbal stem cell niche and some of its constituent elements (LESCs, limbal epithelial stem cells; CSSCs, corneal stromal stem cells; TAC, transient amplifying cells). Reproduced and adapted with permission of Ashworth et al. (2021) [97].

The corneal epithelium lines the external surface of the stroma and is continuous with the conjunctival epithelium. The limbus is the unique junctional zone (1–2 mm) that separates the corneal–conjunctival boundary and the underlying sclera. The limbal epithelial stem cells (LESCs), which are resident in the palisades of Vogt, the infoldings of the limbus, are physiologically responsible for replenishing the corneal epithelial layer (Figure 3) [79]. Until recently, the accepted location of LESC residence was within the palisades of Vogt; however, current studies in mammalian models (human, murine, rabbit and porcine) have identified two secondary “niche-like” structures. These proposed hypothetical niches, the limbal epithelial crypts and focal stromal projections, were distinguished within the adult limbal zone [80]. The limbal epithelial crypts are distinct radial extensions of five to seven solid cords of epithelial cells from the peripheral end of the palisades of Vogt into the conjunctival stroma or circumferentially into the limbal stroma. The focal stromal projections are finger-like protrusions of the limbal stroma into the limbal epithelium that contain a central blood vessel. The complexity of these “niche-like” structures suggests that the maintenance of the corneal epithelium involves not only the prevailing theorised niche for corneal stem cells of unipotent LESCs, but also the oligopotent LESCs distributed throughout the corneal basal epithelium and the putative LESCs observed at the base of the palisades of Vogt [31,72,81,82,83,84]. LESCs are responsible for the homeostasis of the corneal epithelial cells by renewing and repopulating the central corneal epithelium every 7–10 days through proliferation, differentiation and centripetal migration from the limbus towards the central cornea, where they mature and become stratified wing and superficial, squamous corneal epithelial cells. Moreover, the corneal limbus is the barrier that maintains the transparency of the cornea by preventing the invasion of blood vessels and conjunctival epithelial cells. The activity of LESCs within the limbal niche is modulated by a variety of factors, such as (i) biophysical cues responsible for cell–cell (stromal cells, vascular endothelial cells and melanocytes) interactions, cell–ECM interactions and mechanical properties, and (ii) biochemical cues (soluble factors) that influence the LESC phenotype [85,86]. In more recent publications, mesenchymal stromal/stem cells and a stromal stem cell population close to the limbus were identified as two populations and termed collectively as the “limbal niche cells” for their close contact. These corneal stromal stem cells (CSSCs) are multipotent cells in the superficial stroma that have the ability to self-renew and are largely observed in an underlying region between the cornea and the sclera. In the palisades of Vogt, the CSSCs are in close proximity to the LESCs, where they are thought to play a vital role in supporting the stem phenotype of the LESCs and their expansion in vivo, as was further demonstrated in studies performed in vitro where a novel method was developed for isolating limbal stromal stem cells to recreate a limbal microenvironment capable of inducing stem cell fate [32,87,88,89].

The corneal endothelium lacks mitotic activity after birth and, therefore, has limited capacity for regeneration. Normally, in such cases, the neighbouring healthy endothelial cells spread out and change shape to cover the gaps left by lost cells, this is clinically known as polymegathism. However, a line of evidence has accumulated over recent years indicating that there is a putative population of endothelial progenitor cells (EPCs) that exhibit stem cell-like characteristics (expression of alkaline phosphatase and nestin) present in the peripheral endothelium (Table 4) [38,73,90,91,92,93,94,95,96,97,98].

### 4.2. Bioengineering the Corneal Stem Cell Niche

The World Health Organisation (WHO) world report on vision stated that of the 2.2 billion people worldwide that have a vision impairment, 4.2 million of those are due to corneal opacities. There are many types of corneal disease; the more common ones include allergies, dry eyes and keratitis, whilst others such as limbal epithelial stem cell deficiency (LSCD), Stevens–Johnson syndrome and ocular pemphigoid can cause distortion, scarring and eventually blindness [106].

Corneal transplantation is a common treatment option; however, (i) there is a donor tissue shortage due to lack of supplies of corneal donors, (ii) significant immune rejection rates, which then require re-grafting, (iii) possibilities of infection, (iv) not all patients are viable candidates and (v) the waiting lists are lengthy [107,108]. Tissue engineering principles have been used to tackle these challenges by designing biomaterials that mimic the different levels of complexity of the cornea that enable the promotion of cell survival, proliferation, differentiation and subsequent corneal regeneration. Various cell types and biomaterials can be implemented to generate engineered cornea tissue with suitable mechanical, optical and biological properties, leading to valuable clinical outcomes [109,110].

Significant efforts have been made to develop substitutes to replace the damaged or diseased corneal tissue via two approaches: (i) a cell-based strategy and (ii) a scaffold-based strategy. Subsequently, researchers have been able to generate viable levels of complexity in engineering cornea tissue equivalents of the epithelium, stroma and endothelium layers [109,111].

#### 4.2.1. Corneal Epithelium

**Figure 4:**
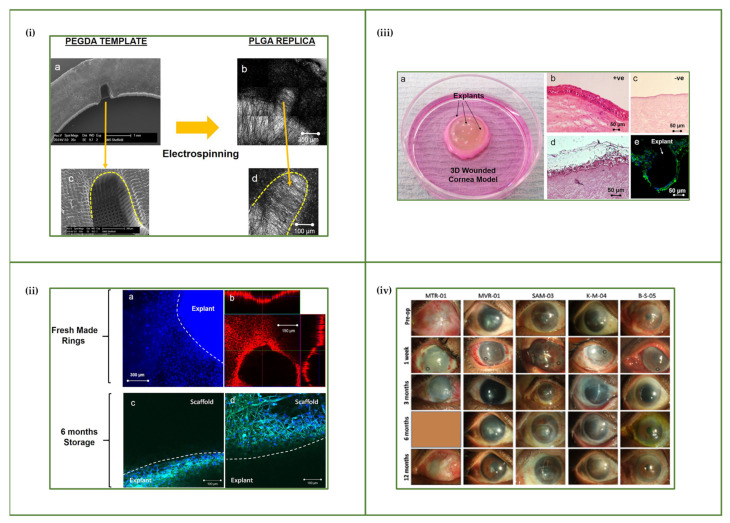
(**i**) The SEM images of the microfabricated pocket electrospun by PLGA onto a PEGPA ring with a horseshoe microfeature. (**ii**) Fluorescence images showing outgrowth of limbal epithelial cells (LEC) from limbal explants cultured on freshly made biodegradable PLGA rings and on rings after 6 months storage at −20 °C (DAPI = blue, Propidium Iodide = red, and positive staining for p63 = green). (**iii**) Demonstrates the rabbit wounded cornea model with panel A illustrating the image of the ring scaffold cultured with the tissue explants. In panel B a fresh rabbit cornea is shown as the positive control; whilst for the negative control the epithelium is removed from the cornea and cultured for 4 weeks as shown in panel C. The H&E image shows the new multi-layered epithelium formed due to cell outgrowth from the limbal explants after 4 weeks in culture in panel D. The immunocytochemistry image shown in panel E illustrates cell outgrowth from a limbal explant (DAPI = blue and positive staining for cytokeratin 3 = green). (**iv**) The first in-man study performed on five patients is shown in the images of the pre-treatment and post-treatment over 1 year. At week 1 the location of explants on the ocular surface are represented with circles and by the 3 months check-up the PLGA membrane was not observed on the ocular surface indicating complete degradation. Reprinted with permission from Ortega et al. (2014) for (**i**–**iii**) and Ramachandran et al. (2021) for (**iv**) [114,116].

The feasibility of harvesting corneal epithelial stem cells on human amniotic membrane (hAM) to bioengineer a corneal surface replacement was demonstrated by Schwab et al., which pointed towards opportunities to tackle difficult ocular surface diseases such as LSCD [112]. However, although it has several favourable properties, the use of hAM clinically has been contended due to shortcomings in the availability of the tissue and the fact that it does not exactly replicate the LSCN. Researchers, therefore, have been attempting to reconstruct the LSCN through man-made means. Electrospinning is an effective and suitable technique for fabricating 3D membranes since it provides matrices that have similar structure to the natural ECM, large surface area-to-volume ratio and high porosity [113]. Consequently, this has led to researchers at the University of Sheffield working with the LV Prasad Institute in India over the past 8–10 years to develop a cost-effective approach for treating corneal disease by designing poly(lactic-co-glycolic) acid (PLGA) electrospun membranes that are biocompatible and present mechanical properties that approach the values shown by native AM. Ramachandran et al. mimicked the LSCN by developing microfabricated pockets (micro-pockets), PLGA 50:50 fibre constructs that contained a U-shaped micro-feature within the membranes, thus providing a protective microenvironment for the limbal explants, (Figure 4i–iii). Subsequently, studies of the micro-pockets in rabbit eyes were conducted and the toxicology results revealed that the micro-pockets did not elicit any topical or systemic toxicology in the rabbits [114,115]. A first in-human study was performed to assess the safety of the PLGA membrane as carriers for limbal tissue explants. The proof-of-concept demonstrated by Ramachandran et al. followed five patients over 1 year after surgery and observed (i) complete degradation of the PLGA membrane by 8 weeks post-transplantation, (ii) epithelium regeneration by 3 months, (iii) no infection or inflammation and (iv) any complication or limitation in vision improvement was found to be not attributable to the PLGA membrane. These results suggest the PLGA membranes are a promising replacement of the hAM for regeneration of the corneal surface in the treatment of LSCD (Figure 4, iv) [116]. They further reported in Paterson et al. that by modifying the membranes by chemical means, either by changing the solvent systems or plasticizer used, fundamental hAM mechanical properties could be mimicked [117].

These results demonstrate the substantial progress made in the research towards developing a commercially available and suitable substitute for hAM in corneal transplantation. However, crucial fine-tuning is required in the biomechanical properties of the PLGA membrane which will have impact on the final product when it comes to (i) storage and packaging, (ii) accessibility and handling by surgeons, (iii) pre- and post-treatment in cases of recurrence of pannus and LSCD in patients and (iv) the use of fibrin glue to hold the limbus explants on the PLGA membranes. Future actions that would be interesting to see is how the newly chemically modified membranes (solvent and/or plasticiser) integrated (i) with vs without micro-pockets, (ii) with vs without fibrin glue, (iii) limbus explants or dissociated limbal cells and (iv) patients’ own limbus explants vs donor explants would perform in an in vivo study.

**Figure 5:**
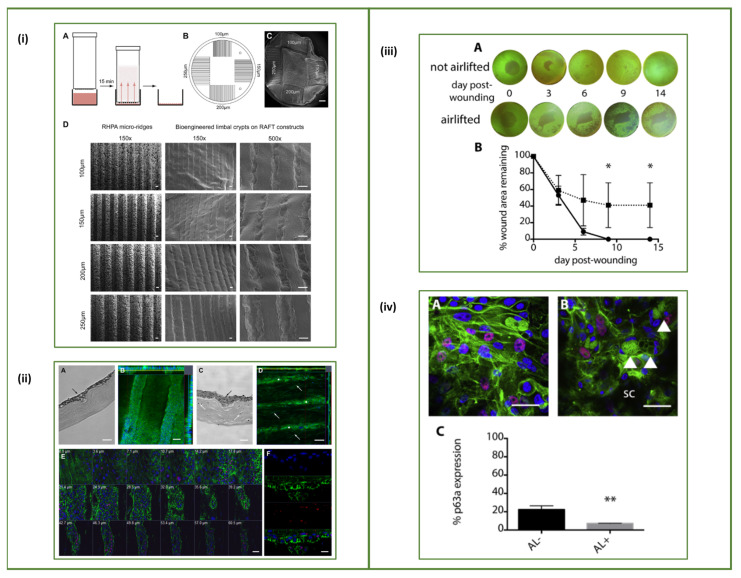
(**i**) Shows the production process of bioengineered limbal crypt (BLC)-containing RAFT constructs using ridged hydrophilic porous absorbers (RHPAs). The SEM images of the four different topologies (protruding micro-ridges of variable depth) on the RAFT constructs. Scale bars C, 1 mm; D, 100 μm. (**ii**) The representative orthogonal confocal images of human corneal epithelial cell line filled BLCs stained with phalloidin (green) and DAPI (blue) illustrate the HLE cells in crypts (white stars) and HLF cells (white arrows) within the RAFT construct as shown in D. In panel E confocal Z-stack images of HLE cell filled BLCs and HLF cells within the RAFT construct are shown as a gallery view (DAPI = blue, Phalloidin = green, and p63a = red). Subsequently, panel F demonstrates the confocal line scan image of the HLE cell filled BLCs (DAPI = blue, Phalloidin = green, and p63a = red). Scale bars panels A and C, 50 μm; B 100 mm; D, 200μm. (**iii**) The effect of airlifting on hLE function after forming a wound on RAFT tissue equivalents. The wounds were imaged using Fluorescein staining on days 0, 3, 6, 9 and 14 post-wounding. Quantification of wound areas on hLE wounded either on non-airlifted (+) or air-lifted (−) RAFT TEs was performed using Image J by measuring the averages ± standard deviation from 3 separate experiments. *p < 0.05 compared to % wound area remaining on non-airlifted RAFT TEs at the same time point. (**iv**) Shows the effect of airlifting on hLE phenotype following wounding and re-epithelialisation. Panel A and B respectively demonstrate the non-airlifted RAFT TEs or the airlifted RAFT TEs at 14 days post-wounding, when the wound had closed (DAPI = blue, Phalloidin = green, and p63a = red). Furthermore, the “sc” denotes the area of hLE sub-confluency on the RAFT TEs that were wounded after airlifting and the white arrows indicate vacuole formation. Scale bars are 50 mm and the images are representative of 5 fields of view at *n* = 3. Quantitative analysis was performed with Image J on the p63a expression of the non-airlifted (AL−) and airlifted (AL+) RAFT TEs. The data presented are averaged over *n* = 3 with ± standard deviation and **p < 0.01 compared to non-airlifted RAFT TEs. Reprinted with permission from Levis et al., (2013) for (**i**,**ii**) and Massie et al., (2014) for (**iii**,**iv**) [118,119].

Levis et al. took a different approach by using a Real Architecture For 3D Tissue, or RAFT^TM^ system, which is a 3D cell culture system. They created microtopographies of 100, 150, 200 or 250 µm micro-ridges in collagen I hydrogels that mimic the 3D architecture of the limbal crypt using a rapid, one-step process. The RAFT constructs were able to provide support to a mixed population of human limbal epithelial cells (hLECs), thus enabling cell survival, proliferation and differentiation, as well as sustaining a population of cells with stem cell characteristics as observed by the high percentage of p63α positive cells lining the engineered microscale crypts. Ultimately, RAFT proved to be an excellent in vitro model to study cell–cell interaction and cell–material interaction by reconstructing the LSCN (Figure 5i,ii) [118]. Advanced studies were performed by Massie et al. where a 3D RAFT tissue equivalent (TE) was wounded and the capability of hLECs to re-epithelialise the wound area was investigated. The process of wound healing is complex, and the results indicated that migration was the cause of cell confluency on the RAFT TE rather than proliferation. Results further suggested that there may be sufficient function in the 3D RAFT TEs to protect the stroma in vivo and eventually in the clinic to treat LSCD patients (Figure 5iii) [119]. The established RAFT TE was used by Dziasko et al. to study the limbal melanocytes’ interaction with the LESCs in the limbal crypt. Co-cultures of human limbal melanocytes (hLM) and hLECs were established from cadaver corneas as the 2D microenvironment and the RAFT TE was established as the 3D microenvironment. The simplified in vitro model of the LSCN maintained cell support and stem cell characteristics as the immunocytochemical analysis showed the presence of the stem cell markers CK15, p63α and Bmi1 and the absence of CK3 [120]. Subsequently, Levis et al. used the RAFT TE model to further characterise the stem cell (ΔNp63α and Bmi1) expression and extracellular matrix (Laminin β1 and Laminin γ3) presence in the in vitro model, as well as the influence that the 3D topography and hLECs had on the underlying human limbal fibroblasts (hLFs). Their results showed that the configuration of the hLFs was similar to the behaviour (parallel extension of cells and alignment) of the stromal cells in the native LSCN (Figure 5 iv) [119,121].

Conversely to the PLGA membrane, the aim of the RAFT TE was to create an in vitro model to allow researchers to study LESC behaviour in a physiological environment that incorporates the characteristic features of the LSCN, making it a versatile tool. The RAFT technology provides a well-characterised graft that demonstrates the potential to be transplanted in clinical application. The foreseeable next stage would be the pre-clinical evaluation of the grafts established from the RAFT technology in vivo, and if feasible, first in human clinical studies to follow. The information that would be gleaned from such a study would provide a reconstructed native LSCN that does not have the issue of rapid biodegradation of the material, such as that in the PLGA membrane. Furthermore, it would be interesting to use the RAFT technology to compare the wound healing in those that have recurring pannus and LSCD versus those that re-establish a healed cornea (healthy versus diseased grafts).

#### 4.2.2. Stroma

Corneal stromal dystrophy and degeneration, together with stromal scarring due to infection, are the most common corneal stromal diseases, and, as the most complex of the three layers that constitute the cornea, the stroma so far has traditionally been replaced by human donor corneas or artificial corneas [122]. However, artificial corneas are known to cause scarring at the transplantation interface, leading to incomplete (i) cellular repopulation of the graft and (ii) nerve regeneration. Recently, enormous progress has been made in the tissue engineering of corneal stroma that enables cell survival as well as restoring functionality [123,124].

Human embryonic stem cell-derived limbal epithelial stem cells (hESC-LESC) were laser-assisted bioprinted (LaBP) into epithelium-mimicking structures on a base constituted from human sourced collagen I and recombinant human laminin. A bioengineering tactic that could mimic the native corneal tissue was investigated by Sorkio et al. where their 3D bioprinted stromal constructs exhibited cell survival, proliferation as well as differentiation, as demonstrated through their co-expression of Ki67, p63a, p40, CK3, CK15, collagen type I and VWF. Seven days after implantation into a porcine corneal organ culture, the 3D bioprinted stromal constructs attached to the host tissue as a result of cell migration from the layered construct. Further studies are required to determine the feasibility of the approach in vitro and in vivo; however, this first study demonstrates its potential for corneal application [124,125].

The 3D bioprinting approach of stromal constructs provides insight into the characteristics required to re-establish an LSCN to support LESCs that differentiate into functional LECs attached without the use of fibrin glue. The cornea is a layered tissue, so a layered tissue-engineered approach would hope to produce a tissue most closely related to that of the native LSCN. Furthermore, 3D bioprinting facilitates high-resolution printing of parameters that allow the incorporation of simple and complex features that are geometrically well-defined in the same construct, such as those observed in the native niche. Ultimately, this approach has the promise of making medical care more personalised, more cost-effective, faster and accessible.

#### 4.2.3. Endothelium

Fuch’s endothelial corneal dystrophy (FECD) is characterised by a thickening of the Descemet’s membrane and loss of corneal endothelial cells. As corneal endothelial cells (CECs) have an extremely limited proliferative capacity in vivo, when this loss reaches a critically low level, corneal oedema occurs, leading to loss of transparency and subsequent impairment in vision and an endothelial transplant is usually required [126]. In order to alleviate global dependency on donor corneas for corneal endothelial-associated transplantation, Peh et al., among others, developed a corneal endothelial cell-based therapeutic [127]. Nanofabrication techniques have been shown to generate key architecture features to support corneal endothelial cell survival and proliferation. Researchers have noted that a combination of techniques (photolithography, two-photon polymerisation lithography, nanoimprint lithography and electrospinning) would be required to produce multiscale and hierarchical structures that would have the ability to biomimic the complexity of the in vivo corneas [128].

Currently, all the attempts to bioengineer the corneal endothelium have been to create a mature endothelial graft rather than to reconstruct an assumed endothelial progenitor/stem cell niche. Whether a corneal endothelial stem cell niche even exists is still under debate, with Sie et al. proposing the summarised table for various corneal endothelial stem cell niches (Table 4) [95]. Furthermore, one of the future challenges lies in designing tissue substitutes that provide the characteristics of the Descemet’s membrane (DM), as studies have shown that the DM is crucial as a substrate in damaged corneal endothelium (CE) regeneration [127]. This has led to researchers bioengineering constructs that are able mimic the DM properties such as (i) physiochemical properties, (ii) 3D architecture and (iii) mechanical properties in order to facilitate the support of functional corneal endothelium as that in the in vivo cornea [78].

A similar microstructure to that of the native DM was reconstructed using two-photon lithography by Gutermuth et al., where they cultured skin-derived human mesenchymal stem cells (hMSCs), which had a neural crest origin in accordance with naturally derived human CECs. Their results demonstrated that the physical cues of the microenvironment influenced the hMSCs to differentiate into functional CEC-like monolayers which were assessed through gene expression (zonula occludens, ZO-1; sodium/potassium-ATPase; paired-like homeodomain 2, PITX2; collagen 8, COL-8) using polymerase-chain reaction (PCR) and by observing the characteristic polygonal morphology of native CEC monolayers. Thus, presenting an innovative and promising approach in regenerating a functional CEC monolayer from hMSCs [129].

Thus far, a combinational approach has been taken with scaffolds that are 2D (hydrogels and/or non-hydrogels) and 3D (bioprinting and/or lithography). Unfortunately, these approaches have not yet reached first in-human studies for the corneal endothelial emphasising that considerable work is still needed for the bioengineered construct to be regulatory compliant and successful in the clinic.

#### 4.2.4. Corneal Innervation

As the most densely innervated tissue, the state of the corneas’ health is dependent on the epithelial and stromal nerve organisation. Much of the research has been focused on the tissue engineering and regenerative medicine of the cornea; however, little advancement has been made in understanding and mimicking the in vivo stromal–nerve interactions [130]. Wang et al. presented the study of mimicking the innervation of the in vivo cornea by encapsulating the corneal epithelial cell layer with that of a corneal stromal cell layer and seeded dorsal root ganglion (DRG) neurons in a 3D silk protein-based co-culture system. Thus, demonstrating their study influenced the survival and alignment of the cells, improving neuronal extensions through the tuneable 3D protein scaffold and creating non-animal models that could be used to research corneal physiology, disease intervention and drug development [131].

Damage in the endothelium is one of the main reasons for corneal transplantation; however, more focus is required to establish an engineering solution that is able to replicate all of the three cell layers and two membranes. A bioengineering approach must be developed that facilitates a 3D system that would be able to maintain the populations of stem cells, promote differentiation when required, maintain the function of cells such as the pump/leak function of the endothelium, as well as encourage innervation throughout the layers.

## 5. Conjunctiva

**Figure 6:**
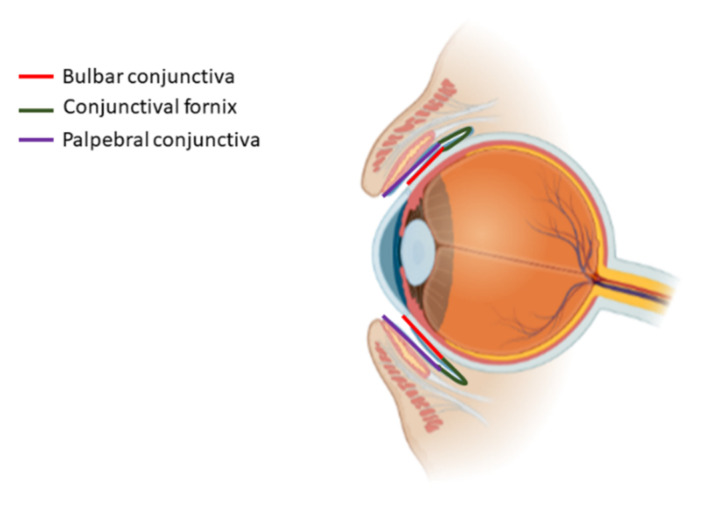
Schematic diagram of the conjunctiva showing its constitutive parts: bulbar conjunctiva (red), conjunctival fornix (green) and palpebral conjunctiva (purple).

The conjunctiva is a thin, translucent membrane that covers the posterior surface of the eyelids and the anterior surface of the sclera, spanning from the eyelid margin to the limbus. This highly vascularised tissue produces the mucin component of the tear film that lubricates the eye [132,133]. Furthermore, the conjunctiva forms a barrier that prevents microorganism invasion and contributes to the immune defence [133]. The conjunctival tissue is divided into three parts: the palpebral, which covers the posterior surface of the eyelids; the bulbar, which covers the anterior sclera; and the fornix, which is the fold lining the cul-de-sac formed between the palpebral and the bulbar parts (Figure 6) [132,134,135]. In all of them, this tissue is composed of a non-keratinised stratified epithelium and a vascularised stroma that is separated from the epithelium by a basement membrane [134]. The conjunctival epithelium is covered with microvilli and contains goblet cells (5–10%) and stratified squamous non-goblet cells (90–95%) [135,136]. While goblet cells secrete gel-forming mucins, those produced by squamous cells are membrane-bound [136,137]. The epithelial cells located at the apical side are interconnected via tight junctions that form a permeability barrier [135]. Melanocytes, which protect against the carcinogenic effects of ultraviolet light, can be found in the basal layer of the epithelium [27,134]. The stroma is formed of vascularised and innervated connective tissue. It contains the conjunctiva-associated lymphoid tissue (CALT), which acts as an antimicrobial defence in conjunction with the lacrimal gland and the efferent tear duct [134,135].

### 5.1. The Conjunctival Stem Cell Niche

It has been reported that the conjunctiva is a self-renewing tissue with a rapid cell turnover and a stem cell niche that replenishes the conjunctival epithelial cells that are continuously lost [133]. Several regions of the conjunctiva have been hypothesised to contain stem cells: the fornix, the bulbar and palpebral conjunctiva and the mucocutaneous junction at the eyelid margin [138,139,140,141,142,143]. The studies on stem cell marker expression (CK19, ABCG2 and p63) and clonogenic ability conducted by Stewart et al. showed that the medial canthal and the inferior fornix are the areas where human conjunctival stem cells are more abundant [35]. This is further evidenced by the fact that the progenitor cells located in these regions have a higher proliferative ability and life span than the cells that populate the bulbar and palpebral regions, and they also show increased expression levels of S-phase markers [137,144,145]. It is important to note that the medial canthal and the fornix are rich in elements that are characteristic of other stem cell niches, such as goblet cells, intraepithelial mucous crypts, blood vessels, melanocytes and immune cells [143]. With regard to their cell potency, it has been shown that these stem cells may be bipotent, as they differentiate into both goblet and non-goblet cells [139,146]. The supply of vitamin A or collagen and the inclusion of conjunctival fibroblasts in organotypic culture are some of the approaches that have been reported to modulate the growth and differentiation of conjunctival stem cells in vitro [147,148,149].

### 5.2. Bioengineering the Conjunctival Stem Cell Niche

The conjunctiva is susceptible to various diseases, including trachoma, chemical and thermal burns, conjunctival lacerations, autoimmune diseases, mucous membrane pemphigoid and Stevens–Johnson syndrome. These can result in cicatrisation, chronically painful eyes and blindness [35,150]. Some of the approaches employed in the treatment of such disorders and injuries include surgical procedures and the implantation of autologous or allogeneic tissue grafts such as the amniotic membrane. However, these techniques are associated with limitations such as the unavailability of healthy conjunctiva for autologous grafts, the occurrence of immune responses, the loss of goblet cells, microbial infection and the opacification of the affected tissue [150]. Therefore, novel approaches have been developed with the advent of novel bioengineering technologies in order to try and prevent these drawbacks. Given the important role of goblet cells in the functionality of conjunctiva, it is fundamental that these novel engineering approaches ensure the restoration of this cell population, which is frequently compromised after injury [151].

As cell–niche interactions have a direct effect on stem cell survival and function, the presence of a functional niche is key for the success of stem cell transplantation [152,153,154]. However, due to the limited understanding of the conjunctival stem cell niche, very few studies have focused on the development of substrates that mimic this niche in order to support the transplantation of conjunctival stem cells into patients. The use of an appropriate substrate for cell transplantation is fundamental, as it improves the retention of the implanted cells in the target site [155].

One of the most promising techniques for the production of artificial conjunctival microenvironments is 3D bioprinting. This approach allows a high degree of control over the cell density and location in the engineered construct. Furthermore, the use of bioprinting technologies enables the production of structures with dimensions, geometry and mechanical properties that are comparable to those of the natural tissue. The inclusion of biological materials in the bioinks leads to a high biocompatibility and an improved regenerative capacity [150]. These properties make bioprinting a promising technology for the creation of artificial structures that mimic the conjunctival stem cell microenvironment. Zhong et al. made use of this novel approach to produce methacrylated gelatine (GelMA) hydrogel micro-structures loaded with conjunctival stem cells for delivery via subconjunctival injection. The stiffness of the hydrogels was controlled by adjusting the light exposure time. The results showed that the structures that were relatively soft led to the highest viability and maintenance of the conjunctival stem cell phenotype [155]. As the mechanical properties of the conjunctival stem cell niche are not known yet, the authors compared the modulus values of the hydrogels with those of the conjunctival epithelium [35,70,150,156]. As these values were different, the authors hypothesised that the conjunctival stem cell niche may have a high heterogeneity in mechanical strength. Another example of the use of 3D printing technology for conjunctival reconstruction is the synthesis of membranes with the ability to regenerate damaged conjunctival tissue. In this study, conducted by Dehghani and co-workers, polymeric membranes made of gelatine, elastin and hyaluronic acid were produced via extrusion 3D printing. The results obtained from the experiments performed in vitro showed that these structures had a good cytocompatibility and promoted cell adhesion and proliferation. The assessment conducted in vivo confirmed that the epithelialisation was comparable to that achieved with amnionic membrane, which is commonly used in conjunctiva repair [157].

**Figure 7:**
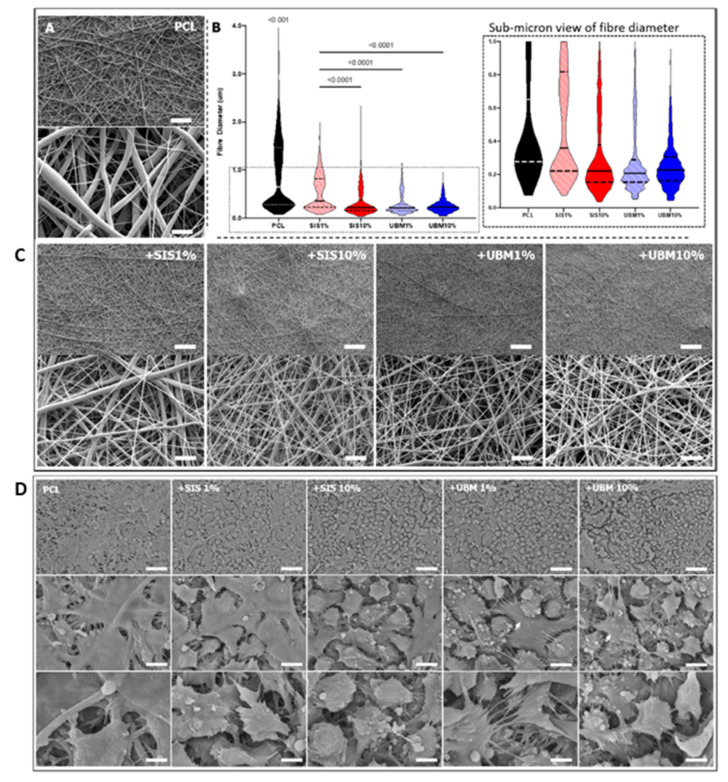
Scanning electron microscopy (SEM) images of electrospun PCL nanofibers without any decellularised ECM (dECM) added to the formulation (**A**) or with 1% or 10% dECM from either small intestinal submucosa (SIS) or urinary bladder matrix (UBM) **(C)**. The fibre diameter decreased significantly with the addition of any dECM, as shown in the violin plot (**B**). Human conjunctival epithelial cells were cultured on the nanofibrous scaffolds. The SEM images showed that the fibres that contained dECM induced the stratification of the cells. (**D**) Reprinted from Bosworth et al. in accordance with CC BY 4.0 [158].

Another technique that has been explored in the production of artificial conjunctival replacements is electrospinning. The nanofibrous scaffolds produced with this method have been shown to promote cell adhesion and proliferation. Furthermore, their architecture facilitates the exchange of nutrients and metabolites. Yao et al. reported the development of electrospun scaffolds made of silk fibroin (SF) and poly(L-lactic acid-co-ε-caprolactone) (PLCL) for conjunctival reconstruction. The rationale behind blending these materials is based on the fact that SF is a natural material that would increase the bioactivity of the scaffold, while the synthetic polymer PLCL would improve the mechanical strength of the resulting material. It is important to note that the SF/PLCL scaffolds supported the phenotypic development of goblet cells. The authors suggested that this could be due to the fibrous and highly porous structure of the membranes, which resembles that of the native ECM and enables the exchange of nutrients [151]. While the incorporation of a protein such as silk fibroin increases the bioactivity of the resulting materials, this does not resemble the composition of the native ECM, which contains a mix of different proteins such as collagen, laminin or fibronectin. Therefore, the addition of a mix of these proteins to the polymer used in the synthesis of nanofibers would lead to the creation of structures that have a biochemical profile that is comparable to that of the ECM. To achieve this, Bosworth et al. produced electrospun scaffolds composed of PCL and decellularised ECM (dECM) [158]. The addition of dECM resulted in the formation of thinner nanofibers with a reduced size distribution and an increased stiffness. It was hypothesised that the decrease in fibre diameter could be attributed to the increased conductivity of the polymer solutions that contained dECM [159]. When used as substrates for the culture of conjunctival cells, dECM-containing fibres showed a complete cell coverage after four weeks, whereas PCL fibres only exhibited a limited cell growth. Furthermore, it was observed that these fibres induced cell stratification, and that this occurred more frequently in comparison with fibres composed of PCL only (Figure 7) [158].

While engineering approaches focus more on the replication of the structure and the biochemical composition of the cell microenvironment, other methods are based on the co-culture of stem cells with other cell types that support their growth and the maintenance of their phenotype. As mentioned above, such cells include stromal fibroblasts, goblet cells, melanocytes and immune cells. The presence of fibroblasts is key for the maintenance of the stem cell population, as they provide growth factors that are fundamental for them. This has been corroborated by experiments performed in vitro that confirmed the role of fibroblast support in the maintenance of an undifferentiated phenotype [160,161,162]. While serum can be used for this purpose during the expansion of cells that are to be transplanted to patients, this poses a risk of transmission of biological agents. Therefore, previous research has focused on the development of serum-free co-culture systems in which fibroblasts are cultured together with conjunctival epithelial cells in order to recapitulate the stem cell niche environment in the conjunctiva. In these conditions, conjunctival epithelial cells showed a high expression of the progenitor cell markers p63α and ABCG2. Furthermore, the reduced number of cells expressing goblet cell markers MUC5AC and PAS in comparison with more traditional culture methods provided more evidence that the co-culture system leads to the maintenance of an undifferentiated state [160].

## 6. Iris

The iris is a circular structure that divides the space between the cornea and the lens into anterior and posterior chambers [17]. It has a diameter of approximately 12 mm and a thickness of 0.8 mm in the proximity of the pupil and 0.2 mm at the periphery [163]. It is functionally comparable to a diaphragm, as it adjusts the size of the pupil depending on the amount of light that reaches the anterior surface of the eye [72,164,165]. This is achieved via contraction or dilation of this tissue, which is exerted by the sphincter and dilator muscles, respectively [166]. Other functions of the iris include adjusting the depth of focus, reducing light aberration in the ocular refracting system and absorbing incoming light [163].

The anterior surface of the iris contains fibroblasts and collagen and is exposed to the aqueous humour. Underneath the fibroblasts lies a layer of melanocytes whose melanin content determines the colour of the iris [163,164]. Beneath the anterior surface, the iris is divided into two layers: a pigmented fibrovascular stroma and a posterior double layer of pigmented epithelial cells. The iris pigment epithelium (IPE) is the layer that is closest to the lens and is involved in the formation and maintenance of the blood–aqueous barrier [167]. It is formed of two layers of highly pigmented cells and has folds that facilitate the iris distension and contraction processes. The melanin content in the stroma and iris pigment epithelium contributes to the iris colour and absorption of light [167]. Finally, the iris root, located in the periphery, is attached to the ciliary body and the cornea–sclera junction, which is known as the iridocorneal angle. This is the area of the anterior surface of the eye where the trabecular meshwork and Schlemm’s canal are located [163,165,168]. A more detailed explanation of the function of these structures is provided in the trabecular meshwork section.

Iris pigmented epithelium cells are highly plastic, and studies conducted in vertebrates have shown their ability to transdifferentiate into cells from different lineages [165]. Two studies that date back to the 19th century demonstrated that IPE cells can regenerate the lens in adult newts [42]. Other work that followed these early findings showed that chick IPEs can form lentoids or neurospheres when cultured under specific conditions in vitro [169]. The differentiation of IPE cells into lentoid structures in these conditions has also been reported in human cells [170]. Furthermore, iris-derived cells from rodents and primates can transdifferentiate into cells that express markers which are specific of photoreceptors [171,172,173]. All this evidence shows that IPE cells have progenitor/stem cell characteristics and a neurogenic potential that is comparable to that of the retinal stem cells located in the ciliary body [43,174,175]. This has been further confirmed by Asami and co-workers, who demonstrated the presence of multipotent cells within the iris pigment epithelium in rodents [42].

IPE cells can also be used to replace retinal pigment epithelium (RPE) cells, as they are both derived from the neural ectoderm. Both cell types share various characteristics such as heavy pigmentation, apical/basolateral polarity, tight junctions, retinol metabolism and phagocytic ability. IPE cell transplantation into the retina requires the use of a substrate for the formation of a cell monolayer, as cells need to maintain a specific orientation. A promising material for the synthesis of such substrates is expanded polytetrafluoroethylene (ePTFE). As this material is hydrophobic, its surface must be functionalised in order to support cell attachment. Nian et al. reported the fabrication of ePTFE coated with fibronectin and n-heptylamine (F-HA ePTFE) and the successful transplantation of IPE cells into the subretinal space of rats by using this substrate [176].

## 7. Lens

**Figure 8:**
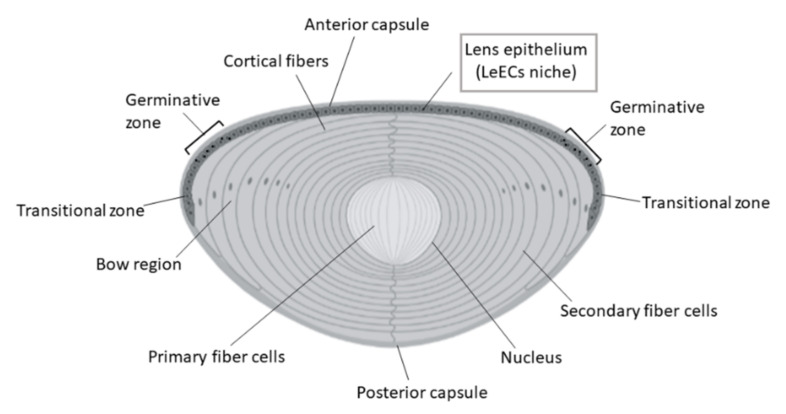
Structure of the human crystalline lens. Adapted from Sharma et al. [186].

The crystalline lens of the vertebrate eye is a transparent, biconvex and elastic structure that is located in the posterior chamber of the eye [177,178,179,180]. It transmits and focuses the incident light onto the retina [1]. This is achieved due to the ability to change its shape in a process known as accommodation. The lens is composed of fibres that are arranged concentrically and a collagenous capsule that protects these structures and contributes to the changes in morphology that occur during accommodation (Figure 8) [178,181,182]. Two types of cells exist in this structure: fibre cells and lens epithelial cells (LeECs) [183]. The latter form a cuboidal epithelium that lies in the anterior capsule and acts as a barrier between the aqueous humour and the lens fibres, hence contributing to the maintenance of the transparency of this tissue [184]. While the central region of the anterior epithelium contains quiescent cells, those located in the peripheral germinative zones are mitotically active [185]. The cells that are formed in the germinative zone migrate towards the posterior side of the lens. Once they move past the equator, they differentiate into fibre cells that stay in the periphery of the lens fibrous mass and displace older fibres towards the interior of the lens. This contributes to the continuous growth of the lens throughout the individual’s life [183].

The vertebrate lens contains high levels of structural proteins known as crystallins. Different types of crystallins, αA-, αB-, β- and γ-, have been detected in this tissue. Higher concentrations of these proteins can be found in the lens fibre cells, leading to the generation of a high refractive index [187]. Furthermore, the expression of different types of crystallin proteins with varying refractive indices leads to the generation of a negative spherical aberration that compensates for the positive spherical aberration caused by the higher refraction of light by the cornea. This increases the accuracy with which the light is focused on the retina [188]. As well as being involved in the maintenance of the refractive index gradient, α-crystallins act as chaperones that prevent the aggregation of other crystallins and unfolded proteins, thus preventing the loss of lens transparency [189,190].

The lens possesses regenerative capacity under specific circumstances where the lens capsule is preserved after trauma. Some authors consider that a subpopulation of the LeECs that reside in the anterior capsule act as stem cells, as they have a slow proliferation rate, differentiate into fibre cells and are recruited after injury of the central epithelium [27,185]. Yamamoto et al. suggested that lens stem cells could be located in the anterior side of the germinative zone [191]. While a specific lens stem cell marker has not been identified yet, they reported the presence of cells that expressed the stem cell marker p75^NTR^ in the germinative zone of the developing mouse lens. However, this becomes undetectable in older lenses. Other stem cell markers such as Msi1 and Lgr4 have been detected in the lens epithelium and differentiating fibre cells [192,193]. Another possible prove of the presence of stem cells in the lens is the identification of side germinative zone which express high levels of various stem cell markers such as ABCG2, p75^NTR^, Nestin, Bcl2, β1 integrin and Sca-1 [185]. As well as the continuous self-renewal and expression of stem cell markers, another requirement that a cell population must fulfil in order to be considered adult stem cells is the ability to regenerate the tissue where they reside. A study conducted by Lin and co-workers suggested that lens epithelial/progenitor cells may also satisfy this criterion. They developed a novel surgical method for cataract removal based on the preservation of the endogenous LeECs in order to regenerate the lens. This minimally invasive procedure allows the regeneration of a functional lens with accommodative ability, a property that is difficult to replicate with current cataract surgery approaches. The efficacy of this technique was proven in both animal models in human patients [39].

Despite all the evidence that seems to confirm the presence of stem cells in the lens, it is important to note that this structure lacks some of the characteristic features of a stem cell niche, such as a high vascularisation or innervation. However, the fact that lens cell division occurs during the entire lifetime of an individual indicates that a stem cell population should be present in this tissue. To explain this, Remington and Meyer hypothesised that the lens stem cell niche may be located outside the lens capsule. Due to its proximity to the lens germinative zone, they suggested that the ciliary body could be the tissue that houses the lens stem cell population. This idea is supported by the fact that cells from the ciliary body can develop lentoids in vitro. The migration of lens stem cells from the ciliary body into the lens capsule could be mediated by the zonular fibres, which integrate with the lens capsule near the germinative zone. Therefore, these could serve as entry points into the lens [183].

Given the inconclusive and scarce evidence with regard to the presence of a stem cell population in the lens, further studies need to be performed in order to confirm it. The knowledge gained from this research would be highly beneficial for the development of novel therapies for lens diseases such as cataract. The current treatment for this disease is a surgical procedure in which an artificial intraocular lens (IOL) is implanted in the capsular bag [194]. Whilst successful in increasing the visual quality of patients, this procedure causes damage in the LeECs and is associated with a significant risk of post-surgical complications such as posterior capsule opacification (PCO). This syndrome results in the development of a secondary cataract as a consequence of the transdifferentiation of LeECs into fibroblastic cells that migrate to the posterior capsule and deposit aberrant extracellular matrix proteins that cause capsular wrinkling and contraction, leading to a loss in transparency [194,195]. An additional disadvantage of the implantation of IOLs for the treatment of cataract is the fact that these implants fail to replicate the fibrous structure of the lens and the accommodation reflex [196]. To try and solve this issue, injectable biomaterials that mimic the structure of the lens have been developed. For them to be suitable for use as lens substitutes, these materials should have a refractive index that is comparable to that of the natural lens (1.42), stable swelling behaviour and comparable mechanical properties [197]. While this has been achieved, such materials have so far not been successful in restoring the accommodation reflex [198]. Thus, further developments are required in order to produce biomimetic bioengineered lens substitutes.

## 8. Ciliary Body

**Figure 9:**
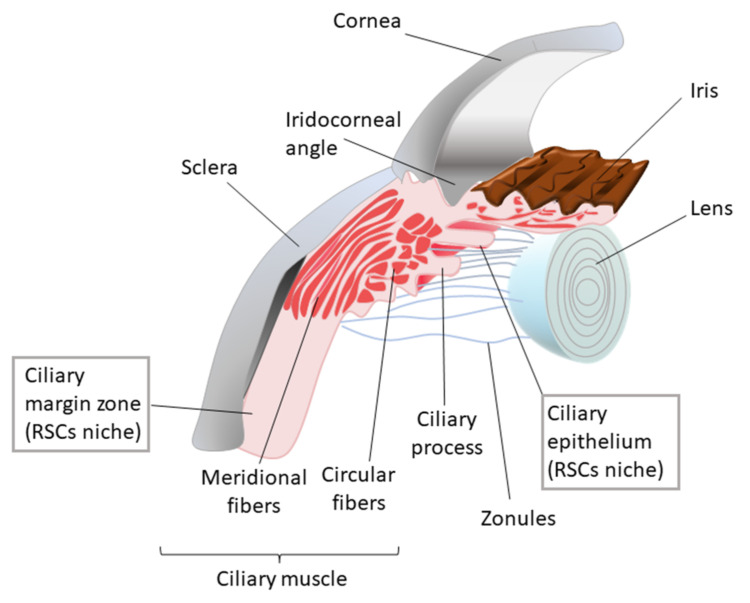
The ciliary body in the human eye. The ciliary epithelium and the ciliary margin zone have been reported to contain retinal stem cells (RSCs). Adapted from Zeiss et al. (2018) [208].

The ciliary body forms a ring of muscular tissue around the interior part of the anterior sclera that extends from the posterior side of the iris to the beginning of the retina, where the ciliary margin zone (CMZ) is located (Figure 9) [199,200]. This structure has a triangular shape in its cross-section and it is linked to the lens via the zonules and to the vitreous internally [168,201,202]. The functions of the ciliary body include the production of aqueous humour and the regulation of both the intraocular pressure and the aqueous and blood flows. It also maintains the immune-privileged status of the anterior chamber and mediates the process of lens accommodation [201,203,204,205,206].

The ciliary body is divided into two different regions. The posterior part, known as the *pars plana* or *orbicularis ciliaris*, is connected to the choroid at the *ora serrata* and also with the vitreous and the periphery of the retina. Its inner surface is flat and contains a high amount of pigment. The anterior portion is known as the *pars plicata* or *corona ciliaris*, which is the area that is involved in the production of aqueous humour. This part of the ciliary body is contiguous to the posterior surface of the iris and has a length of approximately 2 mm. It is formed of 70–100 ridges known as ciliary processes, which increase the surface area for aqueous humour production. These structures are oriented radially around the pupil. Between the ciliary processes, there are spaces where the zonular fibres originate. These structures connect the lens to the ciliary body. Another structure that forms part of the ciliary body is the ciliary muscle, whose contraction opens the trabecular meshwork and the Schlemm’s canal. Therefore, the ciliary body is also involved in the regulation of the aqueous humour outflow through the sclera to the extraocular tissues and ultimately to the lymphatic system [200,201,204,206,207,208,209,210,211].

### 8.1. The Ciliary Body Stem Cell Niche

The ciliary processes are covered by two layers of an epithelium that is formed of secretory cells [201,205,209]. Both the epithelium and the ciliary marginal zone (CMZ) have been reported to contain a proliferative cell population that has been identified by some authors as retinal stem cells (RSCs). The RSCs are a source of retinal progenitors that are recruited during retinal regeneration [212,213]. Studies conducted in *Xenopus laevis* and *Xenopus tropicalis* demonstrated that RSCs from the ciliary margin contributed to retinal regeneration after whole retinal removal [214,215]. These cells, however, may have lower therapeutic potential in humans than epithelial cells due to the reported reduction in neurogenic capacity in higher vertebrates [216]. With regard to ciliary epithelium stem cells, Tropepe et al. showed that these cells have clonal expansion capacity in vitro, they express retinal progenitor markers and they can differentiate into retinal neurons [174,217]. While some studies performed subsequently confirmed these results by differentiating ciliary epithelium stem cells into neurons, retinal ganglion cells (RGCs) and photoreceptors, other investigations failed to replicate these findings [218,219,220]. Cicero et al. showed that although ciliary epithelium cells can proliferate clonally to form spheres and express retinal stem cell markers in vitro, these cells are pigmented, have microvilli and establish epithelial junctions. All these characteristics are distinctive of an epithelial phenotype [221]. Another study that questioned the stem cell identity of ciliary epithelial cells was conducted by Gualdoni et al. The results from this work showed that murine retinal stem cells from the ciliary epithelium cannot differentiate into photoreceptors [222]. However, the self-renewal and proliferative ability of this population of ciliary epithelium cells has been extensively proven, as well as the expression of neural and retinal markers such as Nestin and CHX 10 [174,223,224]. More importantly, it has also been shown that two different cell populations exist within the ciliary epithelium, one that expresses Nestin and another one that is positive for Claudin-1 [225]. The absence of a double-positive population seems to confirm that the ciliary epithelium contains both epithelial cells and neural progenitor cells. However, the expression of stem/progenitor markers in vitro is not enough evidence to confirm the presence of stem cells in a niche as culture conditions and differentiation protocols can affect the cell phenotype in vitro. Thus, it is possible that these are epithelial cells that are highly plastic in vitro and not stem cells [225]. Nevertheless, it is also possible that the negative results observed in some of the aforementioned studies could be due to the sometimes limited of in vitro studies to replicate the processes that take place in vivo. One study that seems to corroborate this theory was conducted by Coles and co-workers. In these experiments, they isolated retinal stem cells from human eyes of different ages and generated spheres that were transplanted into the eyes of mice and chick embryos. It was observed that the progeny of the RSCs integrated into the host tissue and differentiated into photoreceptors [223].

Given the conflicting results obtained regarding to the differentiation potential of these cells, it is essential to conduct functional studies that show whether or not this cell population has the characteristics that are required for them to yield functionally mature cells. A way to assess this is to evaluate their activity after retinal injury. Frøen et al. hypothesised that this cell population would be activated in human eyes that suffer from vitreoretinopathy. While this retinal disease induced cell proliferation in the ciliary epithelium, neural stem cell markers were only detected in a small region of the ciliary epithelium, near the retinal periphery. When performing this same study in mice, the authors detected a Nestin upregulation in the ciliary epithelium. This is of high importance as most of the studies that showed positive results with regard to differentiation of these stem cells into neural lineages were conducted in rodents. Therefore, the contradictory results reported in the literature may be a result of interspecies differences. However, further studies are required in order to confirm this theory [225].

### 8.2. Bioengineering the Ciliary Body Stem Cell Niche

As well as the inconclusive evidence regarding the differentiation potential of the stem cell population found in the ciliary body, the limited availability of these cells would pose a challenge in their use for retinal regeneration [217]. A potential solution to this issue could be the creation of microenvironments that promote the generation of these cells in vitro. The generation of a ciliary margin-like stem cell niche in vitro has been reported by Kuwahara and co-workers. In this study, human embryonic stem cells (hESCs) were used to generate a 3D in vitro model of the neural retina via self-organisation. The differentiation of hESCs into both neural retina and retinal pigment epithelium was achieved by adding specific growth factors at precisely controlled time points to the culture medium. It was observed that a stem cell niche containing ciliary margin-like structures was generated between the neural retina and the retinal pigment epithelium (RPE). The expression of markers that are characteristic of embryonic ciliary margin was detected in this region. It is important to note that the neural retina was polarised and presented the characteristic epithelial morphology that had been previously observed in the periphery of the foetal neural retina. Furthermore, the ciliary margin-like structure was formed in a region of the neural retina that had a close resemblance to the periphery of the embryonic neural retina. The generation of photoreceptors in this culture system has not been reported yet and further studies are required in order to assess the occurrence of this process [226,227,228]. A similar approach has also been reported by Kinoshita et al. for the creation of ciliary epithelium-like structures in vitro. In this case, mouse iPSCs were used for the generation of tissue, which was induced via cell differentiation by adding GDK-3β inhibitor to the culture. The authors suggested that both the time of induction and the addition of other signalling molecules such as BMP and FGF played a key role in the development of this niche. It is of particular interest that this study showed that the ciliary epithelium derived from iPSCs mediated the fluid movement between the two constitutive layers of this tissue. This suggests that the structure was functional and that it could be used to treat ocular hypotony [229].

## 9. Trabecular Meshwork

The anterior segment of the eye is filled with aqueous humour, a fluid that supplies nutrients to the avascular cornea and lens and which maintains the right level of intraocular pressure. This fluid is produced by the ciliary body and flows through the pupil into the anterior chamber. After reaching this point, 40–96% of the aqueous humour is drained into the blood circulation after passing through the trabecular meshwork (TM) pathway [37,230]. This drainage system, also known as the conventional outflow pathway, is formed of the TM, Schlemm’s canal (SC) and the aqueous collector channels. These structures resist aqueous humour outflow, which is driven by a pressure gradient. As a result of this process, an adequate level of intraocular pressure (IOP) is maintained in the eye [37].

**Figure 10:**
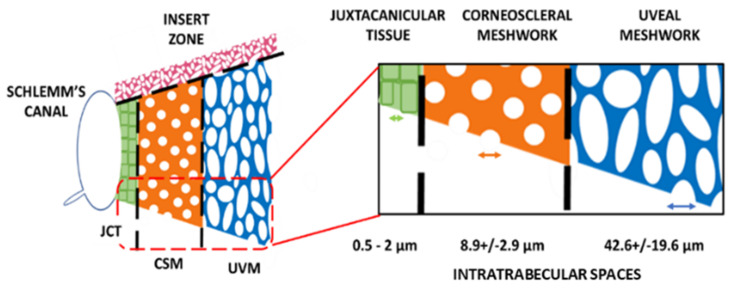
Schematic diagram of the structure of the trabecular meshwork and its four main regions: the uveal meshwork (UVM), corneoscleral meshwork (CSM), the juxtacanicular tissue (JCT) and the insert zone. The insert zone is where progenitor cells reside within the TM. The other zones constitute the “filtering region” of the TM. The dimensions of the intertrabecular spaces in the filtering region of the TM were taken from King et al., (2019) and dimensions of intratrabecular spaces of the JCT are as reviewed in Crouch et al., (2021) [234,241].

Anatomically, the TM is a triangular structure with a length that ranges from 500 to 800 µm and which is located in the anterior segment of the eye, at the iridocorneal angle [231,232]. It contains three structurally-distinct filtering regions: the inner uveal meshwork, which is the closest to the anterior chamber; the corneoscleral meshwork, which is the middle layer, and the juxtacanalicular tissue (JCT), which connects to the SC (see Figure 10) [232,233]. The uveal meshwork is made up of collagen and elastic lamellae in a “net-like” structure of beams around 25.5+/−15.6 µm thick, covered by TM cells, with intertrabecular spaces of around 42.6+/−19.6 µm [234]. The corneoscleral meshwork, contains thicker sections of porous collagen and elastin “beams” or “plates” with smaller intertrabecular spaces around 8.9+/−2.9 µm [234]. The JCT is a connective tissue between the corneoscleral meshwork and the basement membrane of Schlemm’s canal and is mainly ECM surrounding TM cells with intertrabecular spaces between 0.5 and 2 µm [233,235,236,237]. A fourth region of the TM that is not involved aqueous humour filtration is known as the ciliary margin zone (CMZ or insert region), which is believed to be a niche for adult stem/progenitor cells that may act as a reservoir to repopulate cells from the filtering TM region (see below) [238,239]. The width of the insert/transition zone is measured at around 80–130 µm/125–230 µm, with trabecular meshwork beams that extend below the Descemet’s membrane [240].

The specialised cells lining the lamellae of the TM contribute to the filtering ability of this structure by secreting enzymes and phagocytosing cellular debris and reactive oxygen species present in the aqueous humour as it passes through the TM [37]. No singular specific TM marker has been demonstrated and as such a cocktail of known markers and functions are used to characterise TM cells [242]. This is likely due to the differing differentiation, ECM and stiffness of their surroundings. Indeed, the smooth muscle-like contractile nature of the TM is also reflected in some TM cells to be enriched for contractile proteins such as α-smooth muscle actin and myosin and is influenced by agents known to target vascular smooth muscle cells (e.g., nitrous oxide). The heterogeneity of the TM has also been recently demonstrated with scRNA-SEQ, which revealed 10 differing cell populations with two distinct TM populations TM tissue and that not all cells have the same properties [243].

### 9.1. The Trabecular Meshwork Stem Cell Niche

Trabecular meshwork progenitor cells are believed to be primarily but not exclusively located within the CMZ [37,238,244]. Unfortunately, the lack of a specific marker for TM cells also applies to progenitor cell markers. To this end, several studies have demonstrated the presence of putative stem cell markers in cells populating this region, including ABCG2, Notch1, OCT-3/4, ankyrin G and mucin 1 [37,244,245,246, 247]. However, the expression of SC markers tends to be enriched rather than specific to that region of the TM only. Furthermore, depending on the isolation method, there appears to be a heterogeneous population of TM progenitors, with some cells expressing TM cell markers (such as AQP1, MGP, CHI3L1 or TIMP3) while some do not [37]. Whilst the TM progenitors are derived from the neural crest, their plasticity allows them to display different phenotypes (and different gene expression profiles) depending on their surrounding ECM environment. This is mirrored in the plethora of different culture techniques which have demonstrated enrichment for either neuroectoderm or mesenchymal stem cells with TM stem cells that expressed the characteristic markers CD73, CD90 and CD105 [248].

### 9.2. Bioengineering the Trabecular Meshwork Stem Cell Niche

Understanding the pathological changes in the TM is paramount to current and future bioengineering approaches to replicate an artificial TM for medical research or therapy. Glaucoma is the most common cause of irreversible blindness worldwide. Patients suffer from progressive vision loss due to axonal damage and atrophy of the optic nerves [249]. A rise in IOP due to increased aqueous humour outflow resistance at the TM, damages the delicate tissue where nerve axons leave the eye, known as the lamina cribrosa [250,251]. In glaucoma, as TM cells die, the trabecular beams that they once covered can fuse together and cause a thickening of elastic fibres and increased TM stiffness [249,252,253,254]. Last et al. reported an increase in stiffness of the TM from 4+/−2.2 kPa to 80.0+/−32.5 kPa using AFM measurements [255]. Along with a rise in stiffness, glaucoma comes with increased ECM protein “plaque” accumulation such as collagen IV and fibronectin as well as changes in matrix metalloproteinase (MMP) activity [256,257,258].

Trabecular meshwork cells respond to their environment, with changes in stiffness causing changes in gene expression and also cell stiffness [259,260]. Healthy TM cells cultured on cell derived matrices from glaucomatous TM cells (GTM) became stiffer and displayed genetic phenotypes similar to GTM [260]. Therefore, creating an artificial environment that mimics a healthy TM is important in encouraging normal cell behaviour [260]. The manipulation of the environment to mimic glaucomatous TM conditions may also provide a useful tool for studying GTM behaviour.

Due to its complex architecture, creating a bioengineered TM in vitro poses a major challenge [261]. To mimic the TM, it should be formed of multiple layers, mimic the dimensions of the structure and the stiffness, and replicate the porosity gradient found in the natural TM to ensure the biofunctionality of the artificial analogue. A bioengineered TM should also integrate hTM cells and their biochemical and biophysical signals with the biomaterial. Thus, the material used in this process should have similar mechanical properties to the native tissue and must possess the ability to support cell growth, tissue integration and ECM production, resulting in a bioengineered tissue capable of interacting with other ocular tissues in order to maintain a homeostatic environment [261].

Peptide hydrogels have been used to create artificial scaffolds able to repair the TM and to be used as in vitro models to test the efficacy of drugs as glaucoma treatments [260]. Waduthanthri et al. developed an artificial 3D-TM by synthesizing shear-thinning pep-tide–hydrogel scaffolds that were seeded with human TM cells. This system could serve as a 3D in vitro model of the TM and potentialy as an injectable TM implant. The shear-thinning behaviour of this scaffold ensured its injectability, demonstrated qualitatively by injecting it through a 31-gauge surgical needle and via oscillatory rheology. Both cellular and acellular gels possessed shear-thinning properties. After 7 days of culture on these gels, the stiffness of the gel was similar to TM at 1.37+/−0.02 kPa after hTM cells secreted their own ECM proteins, collagen IV and fibronectin. Scaffolds supported the adhesion and proliferation and migration of the hTM cells seeded on them, demonstrating biocompatibility at the cellular level [262].

Another approach that has been explored in the development of artificial TM is electrospinning, a technique based on the application of high voltage to a polymer solution injected through a needle in order to generate micro or nanofibers [260]. Electrospinning is advantageous as it allows production of nanofibers similar in size to that of TM beams [260]. Izaguirre et al. reported the fabrication of “electrospun” porous membranes made of polycaprolactone (PCL) in order to generate an in vitro human TM model. These structures supported the attachment and proliferation of hTM cells over 15 days and also displayed a similar outflow facility to previous findings in native TM. However, the Young’s modulus of the nanofibrous membranes ranged from 3.9 to 20 MPa, around 1000–5000 times greater than healthy and glaucomatous TM [263]. Other disadvantages of electrospinning scaffolds include the difficulty to produce nanofibers with uniform diameters and controlled pore sizes that match those found in the TM. Thus, other approaches that can overcome these issues would be preferred in the creation of an artificial TM [261].

Photolithography can provide a higher degree of control over the pore size and arrangement in order to mimic the structure of the TM. This technique is based on the use of light and a photomask to synthesise or degrade a polymeric material in a specific geometric pattern [264]. The resolution achievable with this technique falls into the submicron scale, allowing the creation of pores with the same dimensions as those found in the TM [261]. Torrejon et al. used porous membranes made of SU-8 as scaffolds to engineer an in vitro hTM model. These scaffolds were fabricated via photolithography to create pre-defined porosity patterns which were then coated with different bioactive molecules to assess hTM cell behaviour. Deposition of a thin layer of gelatine onto the scaffolds led to higher hTM cell attachment and confluence, and a pore size of 12 µm was found to be optimal for cell coverage. Cells grown on SU-8 expressed hTM-specific genes α-SMA, myocilin and αβ-crystallin and secreted ECM proteins, confirmed by immunofluorescent staining. Finally, the outflow ability of the bioengineered hTM was assessed using a perfusion chamber by maintaining a constant flow rate and measuring the pressure across the TM. Increase in transmembrane pressure of scaffolds seeded with cells offered resistance to flow, while the acellular counterparts did not show this behaviour [265].

## 10. Sclera

Scleral thinning is a characteristic change that is observed in the condition of myopia, which is the most common ocular disorder that affects approximately 2 billion people worldwide and this number is projected to rise at least twofold by 2050. Research has shown both genetic and environmental factors play a crucial part in the cause of myopia where the vitreous chamber of the ocular globe is lengthened causing the retina to lie between the focal planes. This causes refractive error, “short-sightedness” that is the leading cause of visual impairment worldwide [266,267,268,269,270].

The sclera is the white 5/6 parts of the outer coat of the eye that is continuous with the cornea anteriorly. This fibrous outer coat consists of 90% collagen and a mixture of 10% proteoglycans and glycoproteins [17,271,272,273]. The sclera is a dynamic tissue that is responsible for performing multiple functions critical to vision, such as (i) providing a strong framework to protect the delicate intraocular structures, (ii) maintaining a fixed axial dimension by withstanding expansive forces generated by intraocular pressure, (iii) ensuring a stable accommodative function by providing a rigid base and (iv) facilitating appropriate aqueous outflow and access to intraocular structures by protecting conduit for the anterior and posterior vascular and neural pathways [72,274,275,276,277].

The sclera consists of the episclera, stromal sclera and laminafusca, which contains the spindle-shaped scleral fibroblasts. Tsai et al. examined whether the sclera contained mesenchymal stem/progenitor cells and found that scleral stem cells were able to express ABCG2, Six2, Pax6 and Notch1, and had a mesenchymal origin. Furthermore, due to their multipotent nature, these scleral stem/progenitor cells (SSPCs) have great potential to be used in cell-based therapy, for the reconstruction of artificial sclera [271,278] and for the treatment of diseases that cause distension of the sclera, such as myopia [17].

**Figure 11:**
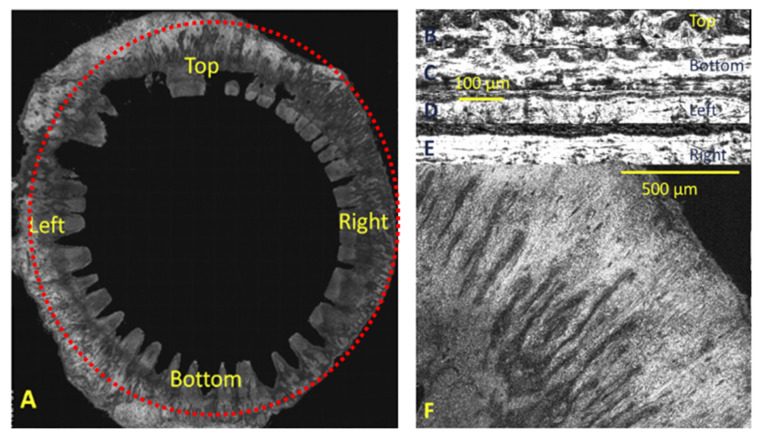
Morphological features of the human limbal zone. (**A**) Entire 360^o^ corneoscleral rim where the corneal button has been removed for keratoplasty. (**B**–**E**) Cross-sections at top, bottom, left and right locations, revealing that in this sample, crypts were visible on the vertical but not the horizontal axis. (**F**) Widefield view of corneoscleral rim portion. Reprinted and adapted with permission from Grieve et al. (2015) [280].

The importance of the biomechanical characteristics of the limbal stem cell niche (LSCN) was reviewed by Eberwein and Renhard as it is an underestimated aspect of the niche. The LSCN is found at the anatomic border of the conjunctiva/sclera and cornea. They highlighted how imperative it is when generating artificial biomimetic constructs such as the cornea, conjunctiva and sclera to keep in mind the mechanical characteristics of the encompassing niche [279]. This notion was further observed by Grieve et al., who investigated the 3D architecture of the LSCN by excising fresh and organ-cultured corneoscleral rims from human donors, and ex vivo samples from pigs and mice to image using full-field optical coherence microscopy (FFOCM). The results demonstrated that all crypt-related architectural features were in the 1 mm width area from cornea to sclera at a depth of 120 µm. They concluded that in order to artificially bioengineer the corneoscleral region, more insight was required in understanding how (i) the behaviour of the species (i.e., nocturnal or diurnal), (ii) the eye position in the species (i.e., front or side of the head), (iii) the presence or absence of eyebrows in species and (iv) how exactly the density of the limbal epithelial stem cells (LESC) have an influence on the architecture of the LSCN (Figure 11) [280].

New approaches for regenerating the scleral tissue are currently being investigated by attempting to make use of tissue components, namely (i) cells, (ii) soluble regulators (cytokines, growth factors), (iii) insoluble regulators (scaffolds) or (iv) a mixture of the above. For instance, treating a thinned sclera can be attempted via cell printing and transfer technology by using the patients’ own cells [281].

Currently, there are very few treatments that target the sclera directly as all strategies so far have been optical or pharmacologic and the long-term negative implications of these treatments on the health of the whole ocular system remain unknown [282].

Researchers must consider the close proximity of the sclera to the limbus as embryologically they originate from the mesoderm. It would be reasonable to reconstruct a niche that incorporates the physical and chemical cues that make up the bordering regions as well as the encompassing cell–cell interactions, as it is these components that maintain cell stemness as well as promote differentiation in homeostasis. Consequently, as this task is so complex, it may explain why most previous research is focused on the biomechanical properties of the sclera rather than attempting to recreate a scleral stem cell niche.

## 11. Future Perspectives and Concluding Remarks

Stem cell therapy has great potential for the cure of ocular diseases, and early success has already allowed clinicians to successfully restore the corneal epithelium [283]. While different types of stem cells are available for use in the clinic, adult stem cells are particularly promising because their use does not raise controversy, as is the case with ESCs. Furthermore, they allow for autologous implantation, with the subsequent reduction in the risk of immune rejection. After isolation from adult tissues, these cells must be cultured in conditions that maintain their undifferentiated state and proliferative potential. This is decisive for the success of stem cell therapies that make use of this cell type. However, the complexity of the natural stem cell niches makes it challenging to study the structural and biochemical factors that play a key role in the maintenance of stem cells. The replication of such environments in vitro could facilitate the researchers’ labour in the elucidation of such factors. Given the heterogeneity and complexity of native stem cell niches, different factors such as ECM-mimicking materials, cells and biomacromolecules are required for the development of such artificial analogues. Furthermore, these components must be processed and arranged in specific ways in order to be able to create biomimetic structures that recapitulate the native architecture of the cell microenvironment. This can be achieved by making use of biofabrication techniques such as bioprinting, electrospinning or photolithography. With regard to the application of such methods for the generation of ocular stem cell niches, several studies have been reported on the development of artificial corneal and conjunctival niches. This contrasts with the limited amount of research outputs available on the bioengineering of lens, ciliary body or iris stem cell microenvironments. Therefore, more studies are required on the replication of such niches in order to increase the success of therapies aiming at restoring them.

Whilst the use of biofabrication techniques has proved successful in the replication of structural features of the cell microenvironment, further work is needed in order to recapitulate the specific cell–cell interactions that take place in native niches. The development of engineered co-culture systems would be highly advantageous in this endeavour. It is also important to consider that the ECM is highly dynamic and that it is through the continuous fine-tuning of its biophysical and biochemical properties that stem cells are regulated [284]. From this, it can be deduced that any artificial analogue should mimic this dynamic environment. The incorporation of stimuli-responsive biomaterials and their functionalisation with bioactive moieties could be a way to achieve this during the development of the next generation of ocular stem cell microenvironments in vitro. It is important to note that this would enable the generation of a self-renewing source of stem cells in such constructs.

As well as the implications that the development of biomimetic stem cell niches would have in ocular stem cell therapy, other areas would also benefit from this advancement. For example, the pharmaceutical industry would benefit from the accessibility to models that can be used as tools for drug screening prior to clinical trials. Regenerative therapies which are not based on the use of stem cells could also benefit from this, as bioengineered constructs can be used as implantable scaffolds for tissue regeneration. Finally, artificial stem cell niches can be used as in vitro models for cancer research, as they can provide an insight into the modulation of cancer progression by the stem cell niche. This could result in the development of more efficient therapies against this disease [19].

## Figures and Tables

**Table 1:** Characteristics of the adult stem cells that populate the different stem cell niches in the anterior segment of the eye.

Niche	Stem Cell Type	Potency	Regulatory Signalling Pathways	Markers	Ref.
Cornea (limbus)	Limbal stem cells	Unipotent	Notch, Wnt, Stat3, IL6, Shh	*Undifferentiated*: TP63, ABCG2, ABCB5, K14, K15, K19, K3/K12, ΔNp63α *Differentiated*: K3, involucrin, connexin 43, KRT12, KRT24, AREG	[28,29,30,31]
	Stromal stem cells	Multipotent		*Undifferentiated*: ABCG2, Nestin, Pax6, BMi1, CD73, CD90, CD166, cKIT, Notch1, Six2 *Differentiated*: ALDH3A1, CXADR, PTDGS, PDK4, CD34 Insignificant expression of fibroblast related genes: αSMA and TCC	[32,33,34]
Conjunctiva (epithelium)	Conjunctival stem cells	Bipotent-goblet and non-goblet cells	NGF-p75-SALL2, Notch and Wnt	*Undifferentiated*: CK19, ABCG2, p63, ΔNp63, Hsp70, KRT15 *Differentiated*: MUC5AC, claudin 10, claudin 2	[35,36]
Trabecular meshwork (insert zone)	Trabecular meshwork stem cells	Multipotent-adipocytes, osteocytes and chondrocytes	-	*Undifferentiated*: ABCG2, Notch1, OCT-3/4, ankyrin G, mucin 1, CD73, CD90 and CD105 *Differentiated*: AQP1, MGP, CHI3L1, TIMP3	[37,38]
Lens (anterior capsule)	Lens epithelial/stem cells	Unipotent	FGF, MAPK	*Undifferentiated*: PAX6, C-MAF, E-cadherin, Sox2, vimentin *Differentiated*: Filensin, CP49, CRYBA2	[39,40,41]
Iris (epithelium)	Iris pigmented epithelium cells	They can form lentoids and neurospheres	bFGF	*Undifferentiated*: Nestin, Msi1 *Differentiated* (*neural*): Map2, TuJ, Gfap, O4 oligodendrocyte marker	[42,43]
Ciliary body (ciliary epithelium and CMZ)	Ciliary epithelium stem cells/Retinal stem cells	Possible differentiation into neurons, RGCs and photoreceptors	Wnt, FGF	*Undifferentiated*: Nestin, CHX 10 *Differentiated* RGCs: Thy1.1, Brn-3b Photoreceptors: rhodopsin Neural: HPC-1 (amacrine cells), calbindin (horizontal cells)	[44,45,46]

**Table 4:** Studies reporting human corneal endothelial progenitors with potential location, identification and differentiated functions. Reprinted with permission from Sie et al. (2020) [95].

Possible Location of Endothelial Progenitors	Methods of Identification	Markers	Remarks	Ref
Not specific corneal endothelium	Sphere-forming assay	Nestin, GFAP, β3-tubulin, αSMA	Dissociated sphere cells showed hexagonal shape and pumping activity; no p75NTR expression.	[98]
Peripheral endothelium (PE)	BrdU labelling and immunostaining	Alkaline phosphatase, Telomerase	Located at the corneal endothelium/TM junction	[99]
	Sphere-forming assay	Nil	PE had a significantly higher percentage of sphere formation, representing precursor density	[100]
	Immunostaining and flow cytometry	Lgr5, Hedgehog pathway markers (SHH, Gli1, Gli2)	Lgr5+ cells were proliferative. Generation of differentiated corneal endothelium and functional assay was not demonstrated	[101]
Central and peripheral endothelium; progenitor-enriched at CE-TM transition region	Immunostaining and flow cytometry	p75NTR, Sox9, FoxC2	Expressed partial properties of neural crest and periocular mesenchyme; differentiated cell sheet had pumping activity by using chamber system and in vivo transplantation to rabbit corneas.	[102]
Whole corneal endothelium of normal and FECD corneas	Colony-forming populations; >80 passages	Pax3, Nestin, Sox9, AP-2β, p75NTR, Sox2, Lgr5, p63, Oct4	Adult corneal endothelium harboured neural crest-derived progenitors capable of perpetual proliferation and formation of endothelial layer exhibiting trans-endothelial resistance.	[103]
Trabecular meshwork (TM)	3D Matrigel culture to activate BMP signalling	AQP1, MGP, CHI3L1, AnkG, Oct4, Sox2, Nanog, ABCG2, p75NTR, FOXD3, Sox9, Sox10, MSX1	TM progenitors differentiated into corneal endothelial cells, adipocytes and chondrocytes.	[104]
TM and transition zone between TM and corneal periphery	Corneal wound model and immunostaining	Alkaline phosphatase, Nestin, Telomerase, Oct3/4, Pax6, Wnt1, Sox2	Wounding activated Oct3/4 and Wnt1 expression as a response to initiate the endothelial repair process.	[38]
Transition zone (inner TZ)	Immunostaining, cell culture	Lgr5, Telomerase, Nestin, Sox2, p75NTR, Pitx2, HNK1	Progenitors projected as multicellular clusters into the adjacent PE. Porcine TZ progenitors differentiated into an endothelial monolayer expressing ZO-1 and ATPase.	[105]

## Data Availability

Not applicable.
